# Tooth loss leads to reduced nutrient intake in middle-aged and older Japanese individuals

**DOI:** 10.1186/s12199-019-0770-3

**Published:** 2019-03-05

**Authors:** Takayuki Kosaka, Momoyo Kida

**Affiliations:** Department of Prosthodontics, Gerodontology and Oral Rehabilitation, Osaka University Graduate School of Dentistry, 1-8 Yamadaoka, Suita, Osaka 565-0871 Japan

**Keywords:** Tooth loss, Nutrition, Socioeconomic status, Gerodontology

## Abstract

Reductions in masticatory function as a result of tooth loss have a negative impact on nutrient intake, decreasing general health. In addition, studies have reported an association between lower socioeconomic status (SES) and both higher numbers of lost teeth and worse nutrient intake status. Nakamura et al. conducted a study to clarify the relationship between number of teeth and nutrient intake status in their paper “Having few remaining teeth is associated with a low nutrient intake and low serum albumin levels in middle-aged and older Japanese individuals: Findings from the NIPPON DATA”, evaluating not only data obtained from a household-based dietary survey but also serum albumin levels as a nutritional biomarker. Importantly, the present study also took into account the individual SES of subjects in the analysis of number of teeth and nutrient intake. The present results show that the trend for poorer nutrient intake with lower number of teeth is more marked among individuals with low SES. It is therefore essential that individual SES is taken into account in efforts to improve nutrient intake and thus contribute to general health through oral health.

## Background

Tooth loss is one of the factors that impair mastication and other oral functions. When teeth are lost due to caries, periodontal disease, or other causes, masticatory function is reduced because it is difficult to pulverize the food with the teeth and form a bolus smoothly. Furthermore, reduced masticatory function causes narrowing the range of options for foods that can be ingested. The resulting changes in dietary habits impact negatively on nutrient intake, resulting in reduced general health. Tooth loss can thus ultimately lead to reductions in activities of daily living (ADL) [1] and quality of life (QOL) [2] and may even affect life prognosis.

Multiple cohort studies from different countries examining the relationship between number of teeth and total mortality have provided evidence that life expectancy increases with retention of multiple teeth. Abnet et al. [3] carried out a follow-up study over 10–15 years of 29,584 healthy Chinese adults aged in their 40s–60s. Subjects were divided into a group with more than the median number of teeth and a group with fewer than the median number of teeth, and a comparison between groups showed that the mortality rate was 13% higher in the group with fewer teeth. Brown [4] carried out a 16-year follow-up survey in America of 41,000 subjects ≥ 18 years old, and the results showed that among subjects ≤ 65 years old at baseline, the mortality rate was 19% for edentulous subjects and only 10% for other subjects.

Lifestyle diseases such as cardiovascular disease and cerebrovascular disease account for a high proportion of deaths, and a possible pathway linking these diseases to number of teeth is the deterioration in nutrient intake as a result of changes in dietary habits. A number of reports have examined the relationship between tooth loss and nutritional intake. Wakai et al. [5] carried out a follow-up survey of 20,366 dentists in Japan from 2001 to 2006 and found that as the number of teeth decreases, intakes of vegetables, carotene, vitamin A, vitamin C, and dairy products are reduced, while intakes of carbohydrates, rice, and confectionery are increased. Zhu and Hollis [6] analyzed 9140 individuals who took part in the National Health and Nutrition Examination Survey in the USA during 2005–2008, and showed that subjects with a greater number of remaining teeth ingested greater quantities not only of minerals and vitamins, but also of proteins, while those with a lower number of remaining teeth ingested greater quantities of carbohydrates.

## Strengths and potential areas of consideration

Nakamura et al. [7] studied the relationship between number of teeth and nutrient intake by evaluating not only nutrient intake status obtained from a household-based dietary survey, but also serum albumin level as a nutritional biomarker. They investigated the relationship between number of teeth and nutrient intake by analyzing data from 2049 individuals ≥ 50 years old obtained from the NIPPON DATA 2010 survey. The results show that those individuals with number of remaining teeth in the lowest 25% show a significantly greater intake of cereals and a significantly lower intake of vegetables and meat than individuals with number of remaining teeth in the uppermost 25%. Thus, individuals with fewer remaining teeth ingest lower quantities of proteins, minerals, vitamins, and dietary fiber as well as greater quantities of carbohydrates and were also shown to have lower serum albumin levels.

In the present study, an analysis was conducted after considering the socioeconomic status (SES) of subjects. Subjects were classified on the basis of high or low SES and a differential analysis was performed, and an even clearer relationship between number of teeth and nutrient intake was found among individuals with low SES.

Tooth loss is believed to be susceptible to economic influences. Seerig et al. [8] conducted a meta-analysis and reported that in the 11 references finally extracted, individuals with low income had a greater chance of tooth loss (odds ratio, 2.52). Kim et al. [9] also carried out an epidemiological study of 7005 individuals who participated in the 2012–2013 Korean National Health and Nutrition Examination Survey, and showed a significantly greater proportion of people with ≥ 20 remaining teeth among the high SES group.

At the same time, the problem of nutritional status among elderly individuals is not only a question of preferences or dietary habits, but also a range of factors such as reduced physical function, psychological aspects, and the living environment. It is therefore essential when evaluating nutritional status not just to ascertain nutrient intake amounts, but also to build up a comprehensive picture of the physical and psychological situation and socioeconomic environment. A meta-analysis by Mayēn et al. [10] extracted 33 papers limited to low- and middle-income countries and showed that individuals with high SES and those living in urban areas generally have healthier dietary habits.

## Conclusions

In the paper by Nakamura et al. [7], the trend for individuals with fewer remaining teeth to have worse nutrient intake was more pronounced among those with low SES. This finding suggests that SES may be intimately involved in the pathway leading from tooth loss to poor nutrient intake. SES of the individual must therefore be taken into account in efforts to improve nutrient intake and promote physical health through good oral health.

## Abbreviations

ADL: Activities of daily living
QOL: Quality of life
SES: Socioeconomic status
